# The Semantic Web—New Ways to Present and Integrate Information

**DOI:** 10.1002/cfg.240

**Published:** 2003-02

**Authors:** Steffen Staab

**Affiliations:** Institute for Applied Informatics and Formal Description Methods (AIFB) University of Karlsruhe (TH) Karlsruhe and Ontoprise GmbH Karlsruhe D-76128 Germany


**Comparative and Functional Genomics**
*Comp Funct Genom* 2003; **4**: 98–103.
Published online in Wiley InterScience (www.interscience.wiley.com). **DOI:** 10.1002/cfg.240

**Conference Review**

### The semantic web — new ways to present and integrate information

Steffen Staab*
*Institute for Applied Informatics and Formal Description Methods (AIFB), University of Karlsruhe (TH), D-76128 Karlsruhe, and Ontoprise GmbH, Karlsruhe, Germany*

**Correspondence to:
Steffen Staab, AIFB, University of Karlsruhe (TH), D-76128 Karlsruhe, Germany.
E-mail:
staab@aifb.uni-karlsruhe.de;
staab@ontoprise.de
Web: http://www.aifb.uni-karlsruhe.de/~sst;
http://www.ontoprise.com*

Received: 30 October 2002
Accepted: 28 November 2002

### Introduction

Ontologies in biology have proved successful in facilitating information access, e.g. by allowing for conceptual browsing of documents or for augmenting information retrieval of documents by semantic structures. So far, however, the predominant view of ontologies has been one where a centralized ontology describes the information structure via which client access to server contents, such as research articles or information about enzymes, is facilitated.

This view is now challenged by the semantic web. The semantic web allows for defining information and ontologies that are interlinked on the web. In analogy to the standard World Wide Web, information sets are not just put somewhere for retrieval, they are also linked, e.g. data in a semantic format such as the Resource Description Framework (RDF [4,10]) may be linked to ontologies described in the same web format [16]. Data that links to the same concept may be discovered by intelligent agents. Thus, in biology we foresee capabilities such as:

1. The retrieval of descriptions of networks of proteins by combining multiple ontologies and multiple data sources — even if the protein names may differ, or if the relevant information may only be found by aggregating information from two different sources.
2. The retrieval of descriptions of gene sequences fulfilling one particular role, rather than a random one out of several that they may act in.
3. The free and effortless re-use of work spent on integrating freely available biology databases; such re-use will be possible by first publishing and then retrieving descriptions of integration efforts in the semantic web.

In this review, we sketch three different semantic web scenarios, listed in an order that reflects their maturity. The semantic portal (SEAL) framework comes first, being operative in different versions since 2000. Second, there is a completed semantic web case study that shows how the work of different information providers may be exploited by several independent parties. Finally, we sketch recent research we have been doing on information integration that lets information providers participate in

Copyright © 2003 John Wiley & Sons, Ltd.

the semantic web, while giving users the freedom to select their own appropriate ontology — and to re-use integration work published by others.

### SEAL — a framework for integration and presentation

The recent decade has seen tremendous progress in managing semantically heterogeneous data, such as are abundant in biology (e.g. in some of the over 500 freely available databases on genomics). Core to the semantic reconciliation between different data sources is a rich conceptual model that the various stakeholders agree on, i.e. an *ontology* [6]. The conceptual architecture developed for this purpose now generally consists of a three-layer architecture comprising (cf. [18]):

1. Heterogeneous *data sources* (e.g. databases, XML, but also data in HTML tables).
2. *Wrappers* that lift these data sources onto a common data model (e.g. the one by Papakonstantinou *et al.* [13] or RDF [10]).
3. Integration modules (*mediators* in the dynamic case) that reconcile the varying semantics of the different data sources.

Thus, the complexity of the integration/mediation task could be greatly reduced.

Similarly, in recent years the information system community has successfully strived to reduce the effort for managing complex contents, e.g. management of extensive websites (e.g. [1,5]). Previously, sparsely structured website management has been organized through process models, redundancy of data has been avoided by generating it from database systems and website generation (including management, authoring, business logic and design) has profited from recent, commercially viable, successes. Again, we may recognize that core to these different website management approaches is a rich conceptual model that allows for accurate and flexible access to data. Similarly, in the hypertext community conceptual models have been explored that implicitly or explicitly exploit ontologies as underlying structures for hypertext generation and use (e.g. [2]).

### Semantic portal

Combining these two approaches, we have proposed SEAL (semantic portal) (cf. [11,15]), a framework for managing community websites and web portals on an ontology basis. The ontology supports queries to multiple sources (a task also supported by semi-structured data models), but beyond that it also includes the intensive use of the schema information itself, allowing for automatic generation of navigational views and mixed ontology and content-based presentation. The core idea of SEAL is that semantic portals for a community of users that contribute *and* consume information require website management *and* web information integration. In order to reduce engineering and maintenance efforts, SEAL uses an ontology for semantic integration of existing data sources, as well as for website management and presentation to the outside world. SEAL exploits the ontology to offer mechanisms for acquiring, structuring and sharing information between human and/or machine agents. Thus, SEAL combines the advantages of the two worlds sketched above.

The SEAL conceptual architecture (Figure 1) depicts the general scheme. Approaches for website management emphasize the upper part of the figure and approaches for web information integration focus on the lower part, while SEAL combines both, with an ontology as the knot in the middle. Thereby, 'presentation' is not restricted to the generation of web pages. It may even include connections to dynamic networks, e.g. in our case we have adapters for connection to the Edutella Peer-to-Peer system (**http://edutella.jxta.org/**).

A (very recently) up and running example for SEAL is given by **http://www.ontoweb.org** — a portal for the ontology community that uses the community to test and verify its own ideas (along similar lines, although with a simpler approach, is the Mathnet initiative, **http://www.math-net.de/**).

A corresponding portal (or several) in the biology community could be driven by a combination of known ontologies (such as the gene ontology). They could serve to integrate information from dedicated databases, as well as from RDF sources known on the web, and present this information in several ways at a community site — allowing for alternative high-precision access with little effort required for maintenance of the portal.

Copyright © 2003 John Wiley & Sons, Ltd.

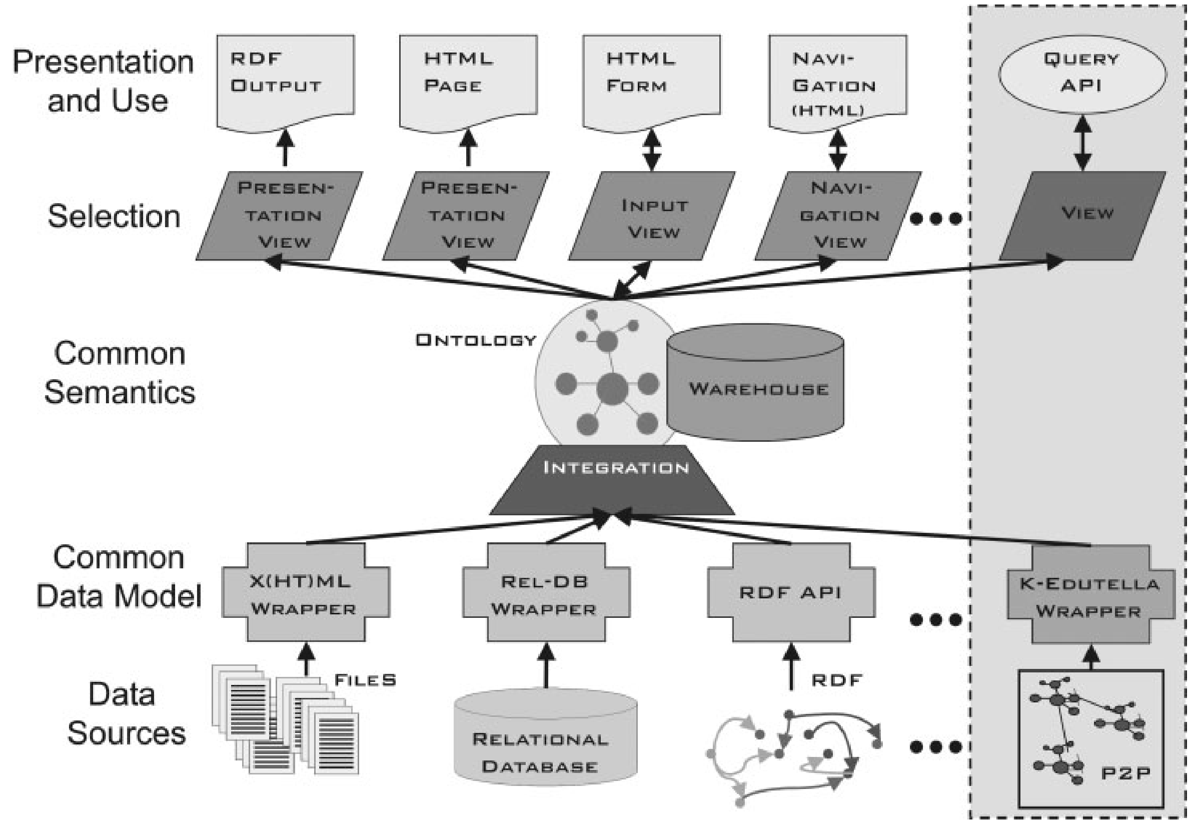

**Figure 1.** SEAL conceptual architecture: gathering from a multitude of data sources for multiple uses in information presentation

### Case study: annotation of papers for ISWC-2002

In spite of its degree of sophistication, SEAL was still geared to being a mostly centralized framework where one ontology and one warehouse have offered centralized control. The semantic web, however, targets at a truly distributed setting. An example for such a setting may be glimpsed from the ISWC-2002 annotation effort: authors of papers at the Semantic Web Conference 2002 were asked to provide an RDF content description for HTML abstracts of their contributions that they could place anywhere in the web. A common procedure, ontology and annotation tool [6] were defined that were not enforced, but which most authors attended to (see **http://annotation.semanticweb.org/iswc/documents.html**). The ontology described papers, researchers, organizations, etc. and their relationships. An example annotation of a paper is shown in Figure 2, which presents the affiliations of two authors.

Annotations were harvested by third parties (Christian Fillies from Semtation, Berlin; Martin Frank and colleagues from ISI USC, Marina del Rey, CA, produced reports from RDF data of ISWC-2002) and used to produce visualizations such as the one depicted in Figure 2.

Thus, the situation was still one where one ontology reigned, but many contributors fed the system on their own, and still other persons exploited them. This is nearly a full semantic web scenario — including far-reaching means for multiple integration and presentation.

### On deep annotation

One of the core challenges of semantic web applications, such as the ones just described, is the creation of metadata by mass collaboration, i.e. by combining semantic content created by a large number of people. To reach this objective, several approaches have been conceived

Copyright © 2003 John Wiley & Sons, Ltd. 

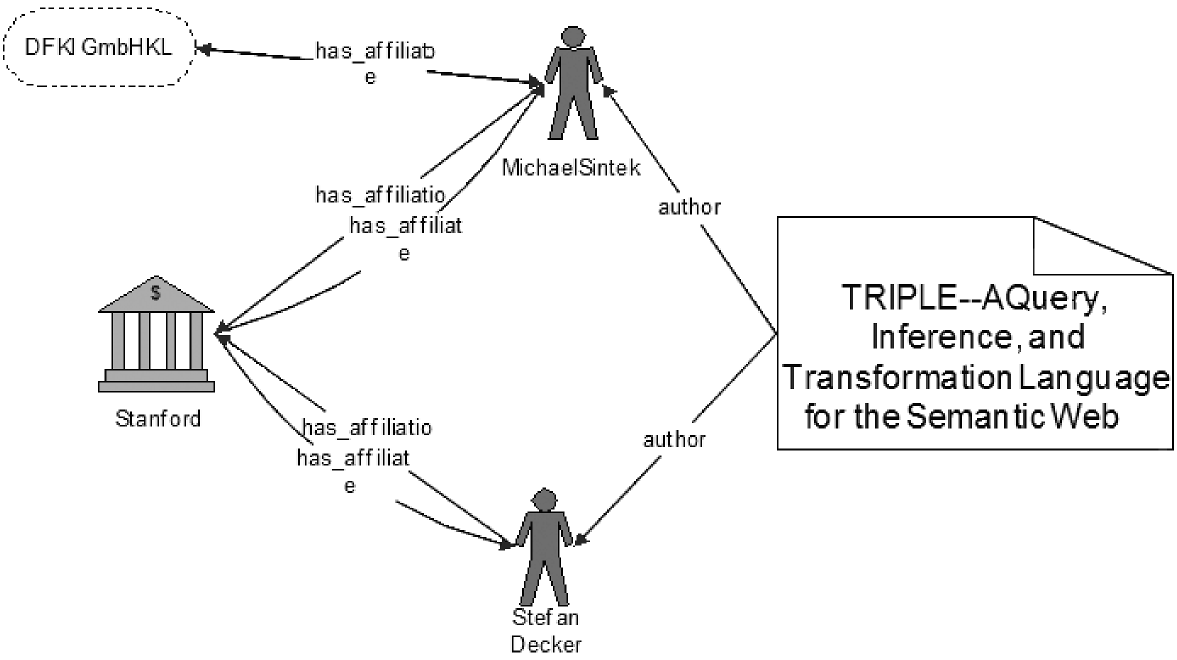

**Figure 2.** An automatically created visualization of semantic relationships (using SemTalk™). Reproduced by courtesy of Semtation GmbH, © Semtation GmbH: **http://www.sc4.org/**

(e.g. CREAM [7,8], MnM [17] or Mindswap **http://www.mindswap.org**) that deal with the manual and/or the semi-automatic creation of metadata from existing information. However, these approaches, in common with older ones that provide metadata, e.g. for search of digital libraries, build on the assumption that the information sources under consideration are *static*, e.g. given as static HTML pages, or as books in a library, etc.

In contrast to this, a large percentage of biology-oriented web pages are not static documents. Rather, the majority of web pages are dynamic. For dynamic web pages (e.g. ones that are generated from databases that contain relations between proteins), it does not seem to be useful to manually annotate every single page. Rather one wants to 'annotate the database' in order to re-use it for one's own purposes. For this objective, approaches have been conceived that allow for the construction of wrappers, by explicit definition of HTML or XML queries [14] or by learning such definitions from examples [9]. Thus, it has been possible to manually create metadata for a set of web pages that are structurally alike. The wrapper approaches come with the advantage that they do not require cooperation by the owner of the database. However, their disadvantage is that the correct scraping of metadata is largely dependent on data layout rather than on the structures underlying the data.

While for many websites the assumption of non-cooperation may remain valid, we assume that many websites about genomics will in fact participate in the semantic web, in order to contribute to progress in biology. Such websites may present their information as HTML pages for viewing by the user, but they may also be willing to describe the structure of their information on the very same web pages, in a semantic web format like RDF. Thus, they give their users the possibility to utilize:

1. Information proper.
2. Information structures.
3. Information context.

A user may then exploit these three in order to create mappings into his own ontology — which may be a lot easier than if the information a user gets is restricted to information structures (as in [12]) and/or only information proper (as in [3]). We define 'deep annotation' as an annotation process that utilizes information proper, information structures and information context in order

Copyright © 2003 John Wiley & Sons, Ltd. 

to derive mappings between information structures [Handschuh S, Staab S, Volz R. On deep annotation (submitted for publication)]. The mappings may then be exploited by the same or another user in order to query the database underlying a website in order to retrieve semantic data — combining the capabilities of conventional annotation and databases.

The process of deep annotation runs as follows:

*Input:* a website driven by an underlying relational database.
*Step 1:* the database owner produces server-side web page mark-up according to the information structures of the database (effectively announcing the contents of his database in a semantic web format!).
*Result:* website with server-side mark-up.
*Step 2:* the annotator produces client-side annotations conforming to the client ontology and the server-side mark-up.
*Result:* mapping rules between database and client ontology.
*Step 3:* the annotator publishes the client ontology (if not already done before) and the mapping rules derived from annotations.
*Result:* the annotator's ontology and mapping rules are available on the web.
*Step 4:* the querying party loads second party's ontology and mapping rules and uses them to query the database via a web service API (application programming interface).
*Result:* Results retrieved from database by querying party.

Obviously, in this process one single person may be the database owner and/or the annotator and/or the querying party. We can align this with an example from biology, as follows. Two providers of information about protein interactions publish their structures, respectively. In database 1 you might find some interactions of protein P, in database 2 you might find some other interactions of protein P. An annotator aligns the sources with his own, or an ontology from some known URL conforming to the RDF schema and publishes the description of how the two sources are integrated. Then, a querying party may exploit the work done by the annotator in order to pose an integrated query to the two databases using the annotator's ontology.

Thus, the semantic web allows for spreading and distributing:

1. Information.
2. Information structures.
3. Information about articulation between ontologies.

Thereby, the semantic web will serve to aid the proliferation of knowledge about scientific databases — ones about biology in particular.

### References

1. Ceri S, Fraternali P, Paraboschi S. 1999. Data-driven one-to-one website generation for data-intensive applications. In *VLDB'99, Proceedings of 25th International Conference on Very Large Databases*, Edinburgh, Scotland, UK; 615–626.
2. Crampes M, Ranwez S. 2000. Ontology-supported and ontology-driven conceptual navigation on the World Wide Web. In *Proceedings of the 11th ACM Conference on Hypertext and Hypermedia.* ACM Press: New York; 191–199.
3. Madhavan DJ, Domingos P, Halevy A. 2002. Learning to map between ontologies on the semantic web. In *Proceedings of the World Wide Web Conference (WWW-2002).* ACM Press: New York; 662–673.
4. Decker S, Melnik S, van Harmelen F, *et al.* 2000. The semantic web: the roles of XML and RDF. *IEEE Internet Comput* **4**(5): 63–73.
5. Fernandez MF, Florescu D, Levy AY, Suciu D. 2000. Declarative specification of websites with Strudel. *VLDB Journal* **9**(1): 38–55.
6. Gruber TR. 1993. A translation approach to portable ontology specifications. *Knowl Acquis* **6**(2): 199–221.
7. Handschuh S, Staab S. 2002. Authoring and annotation of web pages in CREAM. In *Proceedings of the 11th International World Wide Web Conference (WWW 2002)* ACM Press: New York; 462–473.
8. Handschuh S, Staab S, Ciravegna F. 2002. S-CREAM — semi-automatic creation of metadata. In *Proceedings of EKAW 2002.* LNCS, Springer: Berlin; 358–372.
9. Kushmerick N. 2000. Wrapper induction: efficiency and expressiveness. *Artificial Intelligence* **118**(1–2): 15–68.
10. Lassila O, Swick R. 1999. Resource description framework (RDF). Model and syntax specification. Technical report, W3C, W3C Recommendation. **http://www.w3.org/TR/REC-rdf-syntax**
11. Mädche A, Staab S, Studer R, Sure Y, Volz R. 2002. SEAL — tying up information integration and website management by ontologies. IEEE Data Engineering Bulletin: **http://www.research.microsoft.com/research/db/debull/A02mar/issue.htm**
12. Noy N, Musen M. 2000. Prompt: algorithm and tool for automated ontology merging and alignment. In *Proceedings of AAAI-2000.* AAAI Press, Merlo Park, CA; 450–455.
13. Papakonstantinou Y, Garcia-Molina H, Widom J. 1995. Object exchange across heterogeneous information sources. In

Copyright © 2003 John Wiley & Sons, Ltd. 

*Proceedings of the IEEE International Conference on Data Engineering*. IEEE: New York; 251–260.

14. Sahuguet A, Azavant F. 2001. Building intelligent web applications using lightweight wrappers. *Data Knowl Eng* **36**(3): 283–316.
15. Staab S, Angele J, Decker S, *et al*. 2000. Semantic community web portals. *Comput Networks* **33**(1–6): 473–491.
16. Staab S, Erdmann M, Mädche A. 2001. Ontologies in RDF(S) — section on the semantic web (Linköping Electronic Articles in Computer and Information Science). *ETAI Journal* **6**.
17. Vargas-Vera M, Motta E, Domingue J, *et al*. 2002. MnM: ontology-driven semi-automatic and automatic support for semantic markup. In *Proceedings of EKAW 2002*. LNCS 2473, Springer: Berlin; 379–391.
18. Wiederhold G, Genesereth M. 1997. The conceptual basis for mediation services. *IEEE Expert* **12**(5): 38–47.

Copyright © 2003 John Wiley & Sons, Ltd.

